# Psychiatric Consultation at Your Fingertips: Descriptive Analysis of Electronic Consultation From Primary Care to Psychiatry

**DOI:** 10.2196/jmir.7921

**Published:** 2017-08-04

**Authors:** Margaret Lowenstein, Olusinmi Bamgbose, Nathaniel Gleason, Mitchell D Feldman

**Affiliations:** ^1^ University of Pennsylvania Perelman School of Medicine National Clinician Scholars Program Philadelphia, PA United States; ^2^ Marin General Hospital Department of Psychiatry Greenbrae, CA United States; ^3^ University of California, San Francisco Division of General Internal Medicine San Francisco, CA United States

**Keywords:** mental health, primary care, health care delivery, teleconsultation, telehealth, Internet care delivery

## Abstract

**Background:**

Mental health problems are commonly encountered in primary care, with primary care providers (PCPs) experiencing challenges referring patients to specialty mental health care. Electronic consultation (eConsult) is one model that has been shown to improve timely access to subspecialty care in a number of medical subspecialties. eConsults generally involve a PCP-initiated referral for specialty consultation for a clinical question that is outside their expertise but may not require an in-person evaluation.

**Objective:**

Our aim was to describe the implementation of eConsults for psychiatry in a large academic health system.

**Methods:**

We performed a content analysis of the first 50 eConsults to psychiatry after program implementation. For each question and response, we coded consults as pertaining to diagnosis and/or management as well as categories of medication choice, drug side effects or interactions, and queries about referrals and navigating the health care system. We also performed a chart review to evaluate the timeliness of psychiatrist responses and PCP implementation of recommendations.

**Results:**

Depression was the most common consult template selected by PCPs (20/50, 40%), followed by the generic template (12/50, 24%) and anxiety (8/50, 16%). Most questions (49/50, 98%) pertained primarily to management, particularly for medications. Psychiatrists commented on both diagnosis (28/50, 56%) and management (50/50, 100%), responded in an average of 1.4 days, and recommended in-person consultation for 26% (13/50) of patients. PCPs implemented psychiatrist recommendations 76% (38/50) of the time.

**Conclusions:**

For the majority of patients, psychiatrists provided strategies for ongoing management in primary care without an in-person evaluation, and PCPs implemented most psychiatrist recommendations. eConsults show promise as one means of supporting PCPs to deliver mental health care to patients with common psychiatric disorders.

## Introduction

Mental and behavioral health problems are among the most common and costly conditions in the United States [1,2]. Primary care providers (PCPs) play an increasingly large role in diagnosis and management, in part, because of limited access to mental health specialty care [3-6]. Shortages of behavioral health providers have been demonstrated in nearly every US county, and in a national survey, two-thirds of PCPs reported they could not obtain high-quality outpatient mental health services for patients [7,8]. Several solutions have been proposed for these gaps in access, many of which focus on developing more robust systems for mental health care within the primary care setting [9]. These models build on existing evidence that collaborative management between PCPs and behavioral health providers can improve care and access, reduce stigma, and be cost effective [10].

Electronic consultations, or eConsults, are an innovative model of collaborative care developed to improve access to specialty consultation for a variety of physical health conditions. The primary goals of eConsults are to prevent unnecessary referrals for management that could occur in the primary care setting and, in so doing, to decrease wait times for those who require in-person evaluation by the specialist [11]. Improved access to specialists is particularly challenging for systems with provider shortages [12]. Although the format varies, eConsults generally involve a PCP-initiated referral for specialty consultation for a clinical question that is outside their expertise but may not require an in-person evaluation. Most eConsult systems share key features including asynchronous electronic communication between generalist and specialist about clinical questions with documentation within a shared electronic health record (EHR) [12]. Typically, the PCP submits an order for an eConsult with a detailed description of the clinical question, and the consultant responds with recommendations to be implemented by the PCP or, in rare cases, makes the determination that an in-person assessment is needed. Patients are often informed of the eConsult as they would be for a traditional consultation. eConsults are associated with shorter wait times for specialty care, improvement in provider communication and referral quality, and high levels of PCP, specialist, and patient satisfaction [13-20]. eConsults have been successfully implemented in a number of settings, including academic medical centers, the Department of Veterans Affairs, and safety net health systems [21].

eConsults could improve access to mental health care by providing a way for PCPs and psychiatrists to collaborate and expand treatment within the primary care setting. However, acceptability by physicians or patients is not established. In addition, given the nature of behavioral health problems and the importance of the interview in diagnosis, the proportion of eConsult questions that consulting psychiatrists may feel require a face-to-face evaluation is not known. The fact that mental health is often carved out from other medical care creates additional challenges around payment and insurance coverage. Reports on the use of eConsult for mental health are limited but suggest some improvement in PCP perceptions of support for psychiatric diagnosis and treatment as well as access to mental health consultation [22].

Our aim was to test the feasibility of a new psychiatry eConsult program in a large academic health system. We evaluated a consecutive series of the first 50 eConsults to describe the consult questions and responses, assess consult response time and implementation, and determine the rate of conversion to in-person consultation. To our knowledge, this is one of the few studies to describe eConsults for mental health and the first to evaluate consultation content and conversion to in-person referrals.

## Methods

### Study Setting and eConsult Development

The University of California, San Francisco (UCSF) is a multisite urban academic medical center with 8 primary care practice sites and a diverse payer mix including commercial insurance, Medicare, and Medicaid. PCPs include attending physicians, nurse practitioners, and resident physicians. All clinics use a shared EHR (Epic Systems Corp). For more details on the study site, see Multimedia Appendix 1.

The UCSF eConsult program began in 2012 with 8 internal medicine subspecialties; details of the overall program are described in Multimedia Appendix 2 and elsewhere [23]. eConsults were expanded to include psychiatry in October 2014. Psychiatry eConsult protocols were developed through collaborative efforts of PCPs and psychiatrists. Structured consult templates were created for 3 conditions: depression, anxiety, and bipolar disorder. A generic template was created for questions that fell outside the condition-specific list. Full text of the electronic order templates is available in Multimedia Appendix 3. Templates include prompts for PCPs to state a clear consult question and provide relevant historical information, such as medications used, diagnostic instrument results (such as the Patient Health Questionnaire–9), and concurrent substance use. Relevant laboratory studies also autopopulate the electronic template. The consulting psychiatrists consist of 3 attending physicians who review the consult questions individually on a rotating basis. Consultant psychiatrists are expected to respond within 3 business days, and both question and response text become part of the EHR. If the consultant decides that the clinical question requires in-person evaluation, the consult is converted to a traditional referral for an office visit. eConsult payment is supported by the UCSF health system via the University of California Innovations Fund. Specialists are paid on a time-based work relative value unit (wRVU) system, receiving approximately 0.5 wRVUs for a consult. PCPs also receive 0.5 wRVUs in recognition of the fact that the PCP implements the specialist’s recommendation and retains management of the clinical problem. eConsults do not require any insurance authorization, and there is no copayment required from the patient. Further details are available in Multimedia Appendix 2.

### eConsult Analysis

We performed a content analysis on a consecutive series of the first 50 eConsults to psychiatry, occurring between October 2014 and August 2015. We determined our initial sample size based on feasibility. We then analyzed the consults sequentially and found no new themes emerging after analysis of approximately the first 25 consults. We opted to present the entirety of the data in this paper as this adds to the richness of the findings.

Question and response texts were extracted from the EHR with personal identifiers removed. Consult type was determined by the template the PCP selected, with the 4 options being depression, anxiety, bipolar disorder, and a generic template as described above. We examined the content of the generic template and identified a subgroup of consults using this template for questions about psychotic disorders.

We developed a coding framework modeled on prior work classifying eConsult questions and responses and modified by the research team [24-28]. Two independent coders reviewed the text for each eConsult question and response, one internist (ML) and one psychiatrist (OB). First, each question and response was coded as pertaining to one or both of two major domains: diagnosis (identifying the condition) and management (treating the condition). We initially included a third domain for questions pertaining to monitoring, but none of the questions fell into this category. We then defined categories within each domain as below. Each question and response was coded for the presence or absence or each category, and categories were not mutually exclusive.

Within the major domains, we used a hybrid inductive-deductive process to identify categories based on a previously described taxonomy of clinical questions [26] and modified in an iterative fashion to reflect any concepts that emerged during the initial coding process. Categories within the management domain included the following: questions pertaining to medication choice, drug side effects or interactions, navigating the health care system, referral for psychotherapy, and queries as to whether the patient required an in-person evaluation. Specialist responses were categorized in a similar manner. Responses pertaining to diagnosis were coded for the presence of the following categories: interpretation of overall presentation, recommendations for additional testing, and recommendations for obtaining additional history. Within the management recommendations, the following categories were identified: choice of medication, information about side effects, specific medication titration instructions, recommendation for psychotherapy, and recommendation for navigating the health care system.

The two coders analyzed the consult questions and responses independently, and we evaluated agreement using interrater reliability to identify areas of discrepancy and refine the coding structure based on preliminary findings [29,30] **.** Prior to discussion, levels of agreement in the major domains were moderate to high for both PCP consult questions (kappa was 0.4 for diagnosis and 0.66 for management) and specialist responses (kappa was 0.61 for diagnosis and 1 for management). Following the initial independent coding process, the research team met to revise and clarify the coding structure based on these preliminary findings with the final categories derived through consensus process. We then reanalyzed the data using the modified coding structure and any remaining discrepancies were resolved via discussion with the research team.

Additionally, when analyzing questions, we noted whether the PCP had expressed diagnostic uncertainty and whether this was an initial presentation for a patient or the PCP or other provider had tried one or more treatments that failed or resulted in ongoing uncontrolled symptoms. We also noted whether psychiatrist responses included multiple therapeutic options and contingencies or thresholds for starting or adjusting treatment.

### Chart Abstraction

Additional information about consults was obtained via chart abstraction, including completion time by the specialist and implementation of consultant recommendations by the PCP. We considered the recommendation to be implemented if the PCP placed an EHR order for one of the recommended medications, tests, or referrals (including in-person psychiatric evaluation). We also considered a recommendation to have been implemented if the PCP documented taking any of the actions recommended by the consultant in the EHR (including documentation of telephone communications, electronic messages to patients, and clinic notes). Simply documenting the recommendation without any action did not count as implementation. We did not categorize whether PCPs implemented all of the recommendations as many responses included multiple therapeutic options for the PCP to choose between. The time frame for documentation was limited to the 6 months following the eConsult, and assessment of PCP implementation of specialist recommendations was limited to eConsults that recommended a change to existing care plan to be carried out within the study window. Both reviewers extracted implementation data for all 50 consults independently with moderate levels of agreement (kappa was 0.55). We used this initial data to clarify our classification system and then reviewed the data again, resolving any remaining disagreements via discussion with the research team.

### Statistical Analysis

We used descriptive statistics to report patient characteristics, question and response characteristics, and conversion to in-person consultations. Interrater reliability for categorization of consultative focus and implementation of recommendations was determined using Cohen’s kappa statistic.

### Institutional Review

The UCSF Committee on Human Research approved this study.

## Results

### Demographics

The consult patients ranged from 22 to 80 years of age; 54% (27/50) were female, 62% (31/50) white, and 14% (7/50) were African American (Table 1). Of the 50, 19 had commercial insurance (38%), followed by 16 patients (32%) with Medicaid, 10 (20%) with dual eligibility with Medicare and Medicaid, and 5 (10%) with Medicare. This is compared to the general clinic population which is 59% commercially insured patients, 18% Medicaid, 9% dual-eligible, and 13% Medicare. English was the preferred language of 49 of the 50 patients (98%).

**Table 1:** Patient demographics for the first 50 psychiatry eConsults.

Patient characteristics		Total
Age, years, median (range)		48.5 (22-80)
**Sex, n (%)**			
	Female	27 (54)
	Male	23 (46)
**Insurance status, n (%)**			
	Commercial	19 (38)
	Medicare	5 (10)
	Medicaid	16 (32)
	Medicare-Medicaid	10 (20)
**Race, n (%)**			
	White	31 (62)
	African American	7 (14)
	Asian-Pacific Islander	6 (12)
	Hispanic	1 (2)
	Other/unknown	5 (10)
**Preferred language , n (%)**			
	English	49 (95)
	Non-English	1 (2)

### Consult Question Types

Depression was the most common consult template selected by PCPs with 40% (20/50) of consults, followed by the generic template with 24% (12/50) of consults, anxiety with 16% (8/50), psychosis with 14% (7/50), and bipolar with 6% (3/50).

For consults that used the generic template, 37% (7/19) of the questions pertained to psychotic disorders. The remaining 12 of the 19 consults placed using the generic template included less common psychiatric disorders, questions about medical comorbidities, access to care, and others. PCPs expressed diagnostic uncertainty in 22% (11/50) of cases, and for 58% (29/50) of patients, PCPs reported that the patient had persistent or worsening symptoms despite having initiated one or more treatments. PCPs noted prior treatment failures for 80% (16/20) of patients with depression, 88% (7/8) of patients with anxiety, 33% (1/3) of patients with bipolar disorder, 43% (3/7) of patients with psychosis, and 20% (2/12) of patients with other diagnoses.

### Consult Question and Response Content

Textbox 1 contains a sample eConsult question and response using the depression template.

**This sample consult question was coded as pertaining to management, specifically medication choice and drug side effects or interactions. The question also describes prior treatment failure. The response was coded as pertaining to management, specifically including medication choice, conditions for starting/adjusting treatment, medication titration instructions. The response also included multiple therapeutic options.**

**Consult Question Text**

What might be a good alternative antidepressant for this patient?

She is a 64-year-old woman with a history of chronic pain and depression, who has failed multiple selective serotonin reuptake inhibitors and serotonin norepinephrine reuptake inhibitors in past and was recently started on bupropion, but bupropion has not been effective and patient has also had an episode concerning for possible seizure activity. In addition to depressed mood, significant concerns include fatigue, decreased appetite, and significant weight loss.

**Consult Response Text**

Based on review of your summary and patient's chart, my recommendations are as follows:

If the patient is more concerned about pain and its treatment, I would initiate duloxetine. Start at 30 mg PO daily, and pending tolerance, increase to 60 mg PO daily. If improving sleep, increasing appetite, and weight gain is more desirable, mirtazapine would be a good idea. Start at 7.5 mg PO daily, and pending tolerance, increase to anywhere from 15 mg to 45 mg. Sedative effects tend to occur at 15 mg or below, less sedation above 15 mg.

**Sample consult question texts by category (categories were not mutually exclusive).**

**Diagnosis:**

[Patient] presented with concern about someone infecting his urine. He has an anxious affect, insomnia, and these new fixed delusions... [I am] concerned for schizophrenia but patient denies he needs psychiatric care or medication. Not a danger to self or others that I can tell...Do you recommend any diagnostic or treatment options for this young man given that he won't see a psychiatrist?

**Medication choice:**

• Patient with depression and neuropathic pain. Originally on Cymbalta 60 mg daily for neuropathy and depression but depression not well controlled. Started Wellbutrin and has titrated up to 300 mg qAM and 150 mg qPM, but we decreased Cymbalta to 30 mg daily due to increased risk of seizures. Patient has been doing better from a depression perspective but is having more neuropathic pain. Would it be ok to increase Cymbalta to 60 mg daily? Patient without history of seizures or head trauma.

• Patient with anxiety and Generalized Anxiety Disorder–7 Scale score of 12. Functions well during the day but has situational anxiety that is worse, especially flying on a plane. Last primary care provider gave him Ambien, which didn't help. I gave him Ativan 0.5 mg for his last flight. He eventually took 3 of those since it was a turbulent flight. It didn't help. I have given him Ativan 2 mg for his next flight...Is there another med you would recommend for flights or is there an adjunctive med you would recommend?

**Side effects or interactions:**

• Patient with history of episodic anxiety previously managed with as-needed propranolol and lorazepam while giving talks. Her anxiety is less predictable now but she is giving more talks at work and wanted to start maintenance therapy. She had used fluoxetine 10 mg in past with good effect. However when she took one pill this time she developed abdominal cramping, nausea, leg “tremors,” and increased anxiety...I know some nausea and muscle cramping can occur with starting selective serotonin reuptake inhibitors (SSRI) but the tremors and nervousness raise the question of serotonin syndrome for me. Should we try a different SSRI or is it okay to restart fluoxetine?

• Patient with anxiety successfully treated with Zoloft. However, the patient has severe essential tremor and has noticed worsening of tremor on this medicine. In my read of the other anxiety medications (SSRIs, serotonin norepinephrine reuptake inhibitors), it seems like they could all have this side effect. Would there be any medications for anxiety you might recommend that would be less likely to exacerbate her tremor?

**Referral for psychotherapy:**

Any ideas for couples’ therapists for this patient and her husband?

**Navigating the health care system:**

Patient was diagnosed with childhood depression and ADHD...Has some psychotic features such as auditory hallucinations, paranoid thoughts. No history of mania or suicidal ideation. Currently on Lexapro 20 mg daily...Do you have any recommendations as to where he can be seen that can provide him psychiatric services?

**Need for psychiatry or other referral:**

Patient with history of frontal lobe brain tumor status post resection who presents with mood lability...anxiety and depression. Symptoms may be due to location of tumor. Significantly impacting quality of life...Is this something that would best be managed by psychiatry or neurology?

Textbox 2 includes representative question text for each of the major domains and categories.

Most (49/50, 98%) consult questions pertained to management; only in 4% (2/50) was the PCP seeking consultation on diagnosis. Within the management domain, the majority were queries about medication management, including 74% (37/50) of questions about medication choice and 32% (16/50) of questions about drug side effects or interactions. PCPs asked about navigating the health care system in 14% (7/50) of the questions, specifically for assistance helping patients access specialty mental health treatment. For example, PCPs asked for recommendations for services based on insurance status (particularly for patients with public insurance) or for specific types of treatment such as counseling for posttraumatic stress disorder or diagnostic evaluation for eating disorders. Other questions pertained to recommendations about psychotherapy or in-person evaluation.

Psychiatrist responses focused on both diagnosis and management, with 60% (30/50) of responses addressing the diagnosis and all 50 (100%) making recommendations about management (Table 2). Within the domain of diagnosis, psychiatrists commented on the overall clinical picture, offering an impression, differential diagnosis, or working diagnosis for the clinical scenario in 56% (28/50) of responses. Psychiatrists recommended additional diagnostic testing in 6% (3/50) of cases or obtaining additional history in 14% (7/50) of cases. Recommended diagnostic testing consisted of studies, such as thyroid function testing or brain imaging, to exclude organic causes of symptoms. Recommendations for additional history included further clarification of current or prior symptoms, responses to prior treatments, family history, and substance use history.

Management responses also focused on medications, with 72% (36/50) offering advice on choice of medication. Responses also included assistance with specific medication titration instructions in 42% (21/50) of cases, information about side effects in 48% (24/50) of cases, and conditions for starting or adjusting treatment in 34% (17/50) of cases. In 50% (25/50) of responses, consultants offered multiple therapeutic options to be guided by PCP choice or clinical conditions. For example, the consultant might recommend several different medication options depending on specific clinical situations, as seen in Textbox 1. Psychiatrists also offered advice on navigating the health care system in 22% (11/50) of responses and recommended psychotherapy as a primary or adjunctive treatment in 24% (12/50) of responses.

### Recommendations for In-Person Consultation

**Figure 1:**
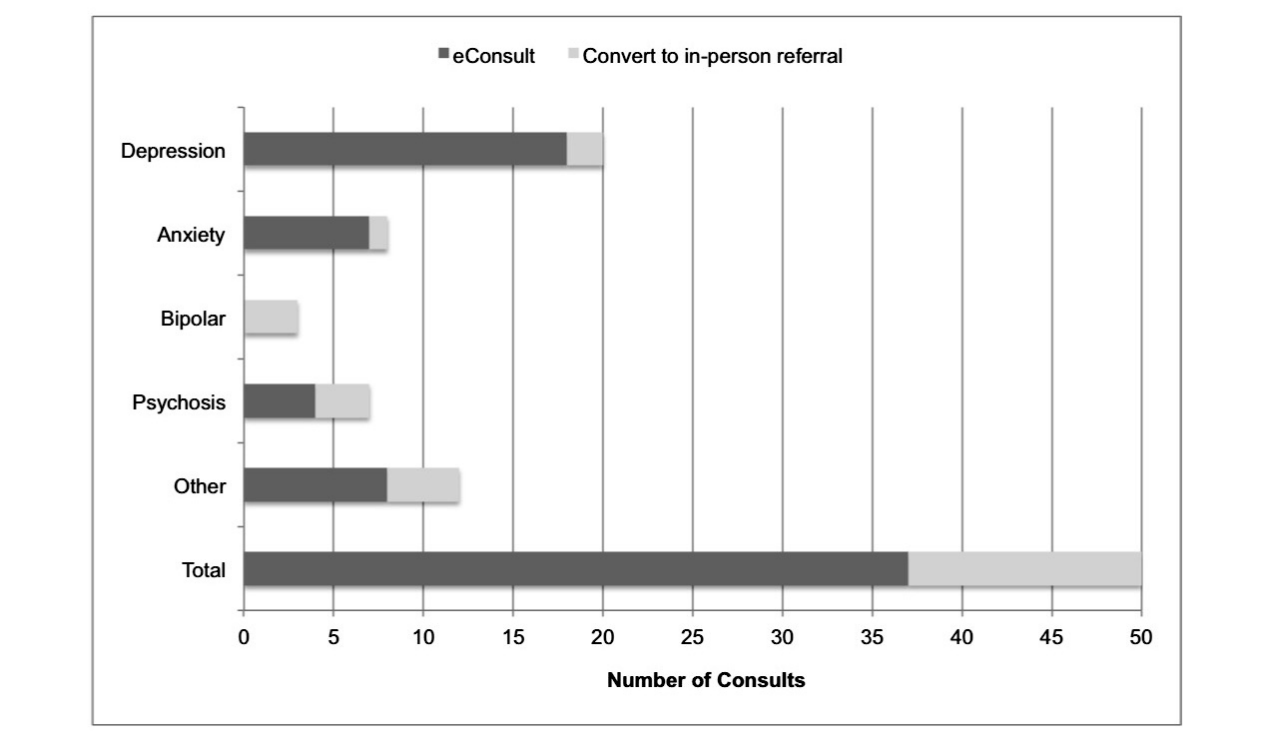
Referral for in-person consultation by consult category.

Psychiatry consultants recommended in-person evaluation or treatment in 26% (13/50) of cases and agreed with ongoing management in primary care for the remaining 74% (37/50) of cases (Figure 1). Recommendations also varied by diagnosis, with fewer of the depression and anxiety consults leading to recommendations for in-person evaluation. Psychiatrists recommended in-person evaluation for all 3 patients with bipolar disorder, 3 of 7 of those with psychotic disorders, and 4 of the other 12 consults using the generic template.

**Table 2:** Breakdown of primary care provider consult question and psychiatrist response text. Question and response texts were coded for 2 domains (diagnosis and management) and for presence and absence of specific categories within these. There could be multiple domains or categories per question or response.

Originator			n (%)
**PCP^a^questions ** **(N=50)**			
	Diagnostic questions		2 (4)
	**Management questions**		49 (98)
		Medication choice	37 (74)
		Drug side effects or interactions	16 (32)
		Navigating the health care system	7 (14)
		Psychotherapy recommendations	3 (6)
		Need for in-person referral	3 (6)
**Psychiatrist responses (N=50)**			
	**Diagnostic recommendations**		30 (60)
		Interpretation of overall diagnosis	28 (56)
		Additional diagnostic testing	3 (6)
		Obtain additional history	7 (14)
			
	**Management recommendations**		50 (100)
		Medication choice	36 (72)
		Conditions for adjusting treatment	17 (34)
		Specific medication titration instructions	21 (42)
		Information about side effects	24 (48)
		Provided multiple therapeutic options	25 (50)
		Recommended psychotherapy	12 (24)
		Navigating health care system	11 (22)

^a^PCP: primary care provider.

### Implementation of Consultant Recommendations

On chart review, PCPs implemented psychiatrist recommendations in 76% (38/50) of consults. The implementation reflected the consultant responses, with the majority of cases consisting of PCPs ordering, modifying, or discontinuing medications. In cases where the recommendations were not implemented, situations varied. For some the patient either improved without intervention or did not follow up. In other cases, PCPs either documented reasons for choosing not to implement recommendations or did not acknowledge the consultant recommendations in the chart. All consults received responses, and the average consultant response time was 1.4 business days, with a median response time of 1 day and range of 0 to 30 days. In comparison, mean and median wait times for an in-person consult in the psychiatry practice during the final 3 months of the study period were 46 days and 38 days, respectively.

## Discussion

### Principle Findings

This study describes the early experience of a large health practice with an innovative program using eConsults from primary care to psychiatry for management of mental health problems. We found that psychiatry eConsults were a feasible way to support PCPs in managing complex psychiatric issues in the primary care setting, with PCPs using the service and frequently implementing at least one of the recommendations. For the majority of patients, particularly those with depression and anxiety, the consulting psychiatrist supported ongoing management within primary care without requesting an in-person psychiatric evaluation and provided a range of strategies that facilitated ongoing primary care–based treatment. In addition, eConsults provided consultation that circumvented common barriers to mental health treatment, including access to treatment providers and insurance coverage since eConsults were available to patient with all payer types without prior authorization from their insurer.

PCPs mainly used eConsults for guidance about treatment, particularly the management of psychotropic medications. PCPs generally made their own diagnostic assessments and used eConsults when confronted with treatment resistance or treatment failure. They also used eConsults to query about systems of care, either navigating the system more broadly or specific recommendations about psychotherapy or psychiatry. Although they were rarely directly asked, the psychiatric consultants often offered suggestions regarding diagnosis, which generally involved summarizing or clarifying the diagnosis. This may reflect a role for eConsults as both management and educational tools.

Psychiatry eConsults share several key features with eConsults in other specialties. eConsults to psychiatry provided timely recommendations for care, with average response time similar to reported values of less than 3 days [21]. Implementation of consultant recommendations was also similar to the 65% to 85% rate described for medical subspecialties within our own academic health system [24,25]. Question content differed somewhat from medical subspecialties [25], with the majority of psychiatric consultations focusing exclusively on management in contrast with the large percent focused on diagnosis and monitoring for medical subspecialties. This may reflect the different nature of psychiatric diagnosis and treatment, which may require more in-person interaction than laboratory testing and imaging. These differences may explain why in-person evaluations were recommended more frequently when PCPs expressed diagnostic uncertainty.

In our study, some PCPs had queries regarding helping their patients navigate the health care system (14%). In particular, this was common for patients who had public insurance that excluded them from obtaining mental health services in our institution due to a carve-out arrangement. System navigation was not a common theme reported for consults for medical subspecialties [25] and may reflect the challenges of accessing mental health care due to fragmentation of both payment and delivery. Nationally, psychiatrists accept insurance at lower rates than other medical specialties, with particularly low acceptance of Medicaid plans [31]. We were unable to track the rates of psychiatry access for our patient population—largely due to insurance carve-outs that require patients to access psychiatric care outside of our health system—but we did find that our study population had a higher proportion of Medicaid and dual-eligible beneficiaries than the clinic population as a whole. PCPs may be using eConsults for patients who are not able to obtain specialty mental health care due to access barriers or for patient refusal. We also found that only one of the psychiatry eConsults was for a non-English speaking patient, as compared to a higher percentage of non-English speaking patients in the clinic as a whole. The literature demonstrates associations between low English proficiency and disparities in access to mental health services [32,33]. Although our small sample size makes it difficult to draw conclusions about these results, our findings may reflect disparities present for eConsults that are similar to more traditional psychiatric consultation.

Given the challenges of meeting growing mental health care demands, there is a need for novel approaches to integrated behavioral health. There is evidence that collaborative management between PCPs and behavioral health providers can provide high-quality care, improve access, reduce stigma, and be cost effective [10]. Other novel technologies including telepsychiatry and other eMental health interventions have been aimed at expanding and extending the delivery of specialty mental health care [34,35]. Like these technologies, eConsult allows for timely provision of care, particularly for those with barriers to access. However, eConsults differ from most described programs in that they target providers rather than patients, allowing for mental health care managed through the PCP in an integrated care model and via a secure EHR. This may help mitigate concerns raised about other eMental health technologies about security, quality, and a lack of a therapeutic relationship between provider and patient as well as worry that patients may defer needed care. Further, an eConsult model has the potential to take advantage of key strengths of the integrated behavioral health models including high levels of adherence and patient satisfaction and reduced stigma [10,36] because they are also based in the primary care setting.

### Limitations

Our analysis has several limitations. First, as a largely qualitative analysis, our sample may not be completely representative of all possible eConsults to psychiatry. Our study was also conducted in a single institution with a robust EHR, and the results may not be generalizable to other institutions with different capabilities. However, we feel our study provides an in-depth look at the nature and scope of PCP questions regarding management of mental health conditions that may be more broadly applicable. Further, our initial interrater reliability was moderate for some portions of our content analysis, although we used this information to identify discrepancies in the coding process, refine our categories, and ultimately create a rigorous coding scheme derived through consensus within the research team. Another important limitation of our analysis is the lack of availability of data for mental health care utilization. Because of separate payment structures for mental health as well as a high percentage of psychiatry referrals outside of our health system, we were unable to track the percentage of eConsults compared to total psychiatry referrals or the impact of eConsult on utilization. We were also unable to evaluate treatment outcomes or compare these with outcomes for traditional care.

### Conclusions

Overall, our data suggest that eConsults show promise as one means of supporting primary care providers to deliver mental health care to patients with common psychiatric disorders, particularly those with depression and anxiety. By extending and enhancing traditional psychiatric consultation within the primary care setting, the eConsult model adds to the burgeoning literature on innovative models for integrated mental health. Future efforts should be directed at evaluating the effectiveness of this intervention in terms of clinical and systems outcomes as well as PCP and patient satisfaction to improve overall understanding of best practices for behavioral health integration.

## Appendix

Multimedia Appendix 1 Study setting.

Multimedia Appendix 2 Development and implementation of eConsult.

Multimedia Appendix 3 eConsult templates.

## Abbreviations

EHR: electronic health record
PCP: primary care provider
UCSF: University of California, San Francisco
wRVU: work relative value unit
