# Supplementary material for: Psychiatric Consultation at Your Fingertips: Descriptive Analysis of Electronic Consultation From Primary Care to Psychiatry
Source: J Med Internet Res. 2017 Aug 4;19(8):e279. doi: 10.2196/jmir.7921 (PMC5562932; doi:10.2196/jmir.7921)
Supplement: Multimedia Appendix 1 [file jmir_v19i8e279_app1.pdf]

Appendix 1: Study Setting

UCSF is a multi-site urban academic medical center with eight primary care practice sites including family medicine, general internal medicine, geriatric primary care, women's health primary care, and a multidisciplinary HIV/AIDS primary care practice. PCPs include attending physicians, nurse practitioners and resident physicians. There are 60 faculty attending physicians; all are general internists, approximately 60% are women, and 70% are full or associate professors. There are 69 Internal Medicine resident physicians who train at the clinic and five nurse practitioners. The resident physicians care for far fewer patients than do the faculty and nurse practitioners.

All clinics use a shared electronic medical record (EHR) (Epic Systems, Inc., Verona, WI). These clinics serve a diverse patient population, with racial/ethnic minorities making up almost fifty percent (21% Asian, 9.6% African American, 8.7% Latino, 13% other). 15% are non-English speaking. Patients age 18-34 years make up 18% of total general medical patients while older adults age 65 and over make up almost 30% of patients (11.5% are age 75 and older). There is a diverse payer mix, including commercial insurance plans (58.7%), Med-Cal (18.2%), Medicare (12.6%), and Medi-Cal plus Medicare (9.1%). Traditional consults are paid for using a fee-for-service model.
