# Supplementary material for: Psychiatric Consultation at Your Fingertips: Descriptive Analysis of Electronic Consultation From Primary Care to Psychiatry
Source: J Med Internet Res. 2017 Aug 4;19(8):e279. doi: 10.2196/jmir.7921 (PMC5562932; doi:10.2196/jmir.7921)
Supplement: Multimedia Appendix 2 [file jmir_v19i8e279_app2.pdf]

Appendix 2: eConsult Development and Implementation

The University of California San Francisco (UCSF) eConsult program began in 2012 and was designed to enable asynchronous consultations between primary care providers (PCPs) and specialists for clinical problems that were unlikely to require an office-based clinic visit. The program is available to all UCSF primary care clinic sites. eConsults use structured templates for commonly referred clinical problems. Templates were developed with input from PCPs and specialists and were embedded into the referral order module of the EHR. They include prompts to articulate a clear consult question and include pertinent history, as well as recommendations for any pre-consultation studies. Specialists are asked to respond within three business days, and both the question and response text become a part of the EHR. If the consultant feels that the question requires in-person evaluation, they convert the consult to a traditional referral for an office visit in that specialty. eConsult payment is described in the text.

Initially, eConsults were available for eight medical subspecialties; the program was expanded to include a number of other specialties including psychiatry starting in October 2014 as described in the text. In total, 10 psychiatry eConsults were placed in 2014 (from October-December), 68 consults were placed in 2015, 60 consults were placed in 2016. Complete results are not yet available from 2017 but there were 20 consults placed as of March 2017. In comparison, during the final three months of the study period, there were between 128 and 176 traditional psychiatry referrals placed per month. However, we were unable to track the rates of in-person visits resulting from these referrals.
