# Supplementary material for: Psychiatric Consultation at Your Fingertips: Descriptive Analysis of Electronic Consultation From Primary Care to Psychiatry
Source: J Med Internet Res. 2017 Aug 4;19(8):e279. doi: 10.2196/jmir.7921 (PMC5562932; doi:10.2196/jmir.7921)
Supplement: Multimedia Appendix 3 [file jmir_v19i8e279_app3.pdf]

Appendix 3: eConsult templates

I am requesting an eConsult from psychiatry for this *** y.o. man with depression.

In the clinical question or current note, please include:

- PHQ-9 score
- History of manic episodes
- Current or past psychotic features
- Concurrent alcohol or substance use disorder (use smartphrases ".alcohol" & ".substanceuse" to help with your assessment)
- Prior treatments, approximate dates, and response

My clinical question: ***

The most current assessment of this problem can be found in the Apex note dated 4/25/2017

The following results are available in Apex (or were recently ordered): TSH, CBC, creatinine, LFTs

----

If this clinical question is deemed too complex for eConsult, please, schedule this patient for in-person consultation. The patient understands that he may receive a phone call from the specialty practice to schedule an appointment.

OR

Route back to me and I will discuss further with the patient.
